# PfCRT and PfMDR1 modulate interactions of artemisinin derivatives and ion channel blockers

**DOI:** 10.1038/srep25379

**Published:** 2016-05-05

**Authors:** Richard T. Eastman, Pwint Khine, Ruili Huang, Craig J. Thomas, Xin-zhuan Su

**Affiliations:** 1 Laboratory of Malaria and Vector Research, National Institute of Allergy and Infectious Diseases, National Institutes of Health, Bethesda, Maryland, USA; 2 Division of Preclinical Development, National Center for Advancing Translational Sciences, National Institutes of Health, Bethesda, Maryland, USA

## Abstract

Treatment of the symptomatic asexual stage of *Plasmodium falciparum* relies almost exclusively on artemisinin (ART) combination therapies (ACTs) in endemic regions. ACTs combine ART or its derivative with a long-acting partner drug to maximize efficacy during the typical three-day regimen. Both laboratory and clinical studies have previously demonstrated that the common drug resistance determinants *P. falciparum* chloroquine resistance transporter (PfCRT) and multidrug resistance transporter (PfMDR1) can modulate the susceptibility to many current antimalarial drugs and chemical compounds. Here we investigated the parasite responses to dihydroartemisinin (DHA) and various Ca^2+^ and Na^+^ channel blockers and showed positively correlated responses between DHA and several channel blockers, suggesting potential shared transport pathways or mode of action. Additionally, we demonstrated that PfCRT and PfMDR1 could also significantly modulate the pharmacodynamic interactions of the compounds and that the interactions were influenced by the parasite genetic backgrounds. These results provide important information for better understanding of drug resistance and for assessing the overall impact of drug resistance markers on parasite response to ACTs.

Malaria, caused mostly by the deadly *Plasmodium falciparum* parasite, is a serious public health burden that still causes an estimated 600,000 deaths and 300–500 million infections each year^1^. As there remains no effective vaccine, treatment in nearly all endemic regions relies on artemisinin (ART) combination therapies (ACTs). An ACT generally combines an ART derivative with a long acting partner drug to maximize efficacy during the typical three-day regimen. Although combined empirically, ACTs typically have synergistic drug-drug interactions that further improve the efficacy of the combination. However, resistance to nearly all antimalarial partner drugs currently in use have been reported, in addition to recent reports of parasites with reduced sensitivity to ART derivatives currently employed in ACTs^2^. A better understanding of how the parasite responds to individual drugs and drug combinations, and how the parasite’s genetic determinants affect the responses, will provide important information for optimal formulations of ACTs and for malaria treatment.

One of the proposed mechanisms of anti-plasmodial activity of ART is the required cleavage of the endoperoxide bridge mediated by heme-derived iron, which generates drug metabolites capable of causing widespread proteome damages that result in parasite death. It has been shown that antimalarial activity of ART is dependent on hemoglobin digestion by the parasite, a process that is required for ART-induced oxidative stress^3^. A fluorescent ART trioxane derivative was shown to rapidly accumulate within digestive vacuole (DV)-associated neutral lipid bodies of trophozoites and schizonts, suggesting that the compound is activated by heme-iron, leading to oxidation reactions that damage parasite membranes^4^. Additionally, various studies have recently begun to elucidate the genetic basis of reduced susceptibility to ART and its derivatives^3^,^5^, and candidate genes or genetic loci associated with altered response to ART have been identified^6^,^7^,^8^,^9^,^10^,^11^,^12^,^13^,^14^. In one study, analyses of ART responses in 34 F1 progeny of the Dd2 × HB3 genetic cross showed that reduced ART susceptibility *in vitro* was a multifactorial trait, linking the response to a locus on chromosome 5 containing the *Plasmodium falciparum* multiple drug resistant gene 1 (*pfmdr1)* and two additional loci on chromosomes 12 and 13, respectively^15^. Additionally, several single nucleotide polymorphisms (SNPs) were associated with *in vitro* IC_50_ values of field isolates’ response to dihydroartemisinin (DHA)^7^. Substitutions of certain amino acids in PfMDR1^13^,^16^ and copy number variation have also been shown to affect parasite response to ART^17^,^18^,^19^. More recently, a molecular resistance determinant (a gene with a K13-propeller domain or K13) that strongly associates with delayed parasite clearance (DPC) *in vivo* has been defined^10^, which was subsequently shown to modulate susceptibility in a ring-survival assay^11^,^14^. Results from the ring-survival assay have been shown to correlate well with estimates of DPC time in patients^20^,^21^. However, due to the short *in vivo* half-life of ART derivatives, the DPC likely represents a drug response phenotype different from IC_50_ values measured *in vitro*. Indeed, many parasite isolates collected from Western Cambodia still had low IC_50_ values (<10 nM), although their IC_50_ values were significantly higher than those from Thailand and South America^7^. It is possible that the differences in IC_50_ values represent background genetic variations that may support the emergence of higher DPC phenotype, including drug transporters such as the *P. falciparum* chloroquine resistance transporter (PfCRT) and PfMDR1 that are known to transport drugs across the parasite digestive vacuole (DV) membrane^22^,^23^,^24^.

A drug resistance phenotype is usually determined or influenced by mutations and/or changes of expression in more than one gene. Inhibition of a protein not only can directly affect its functions, but also can perturb the functions of other genes indirectly through related cellular network. It has been shown that mutations in PfCRT can affect the expression of a specific set of genes, suggesting a complex consequence of mutations in response to drug pressure^25^. To study complex drug resistance phenotypes, genome-wide approaches combining high throughput screenings and linkage/association mapping using genetic cross progeny, field isolates or genetic mutants have been described^26^,^27^,^28^,^29^,^30^. Parasite responses to a large number of compounds have been linked to and/or associated with PfCRT, PfMDR1, or *P. falciparum* dihydrofolate reductase (PfDHFR) after screening progeny from genetic crosses and field isolates against chemical compound libraries^27^,^28^. More recently, piggyBac single insertion mutants were screened against antimalarial drugs and metabolic inhibitors, showing that drugs targeting the same pathway generally had similar response profiles^29^. Additionally, chemical screenings have been employed to predict functional and regulatory divergence of PfCRT^25^ and to show that small molecules associated with similar transcriptional responses also had similar chemical features and/or mode of action (MOA)^30^. Another approach to study genes associated with drug resistance is to pressure parasites to become resistant to a drug and then sequence the genomes of the sensitive wild type and resistant mutant parasites to detect mutation(s) that are likely to play a role in the resistance phenotype^31^.

Large-scale screenings often generate candidate genes and hypotheses that require further experimental verification. Previously, we screened a large number of chemical compounds against 61 field isolates and 67 progeny from the Dd2 × HB3 and GB4 × 7G8 crosses and identified a group of compounds with profiles of parasite response similar to that of DHA, including many ion channel blockers. Our data also showed that parasite responses (IC_50_s) to some of the compounds were linked to PfCRT and/or PfMDR1. To further investigate the interesting observations on parasite responses to selected Ca^2+^/Na^+^ channel blockers, their interactions with ART and derivatives, and the roles of PfMDR1 and PfCRT in drug-drug interactions, here we have performed additional experiments/analyses and showed good correlations in parasite responses to DHA and selected Ca^2+^/Na^+^ channel blockers by plotting data from the chemical screening^28^. We confirmed the interactions of DHA and various channel blockers using isobologram analysis. We then mapped a representative Na^+^ channel blocker (lidocaine) to PfCRT using progeny of a genetic cross and confirmed that PfCRT affected parasite response to lidocaine and lidocaine/DHA interaction using PfCRT allelic exchanged parasites. Similarly, we showed PfMDR1 could affect parasite response to lidocaine and lidocaine/DHA interaction, but only in a parasite line carrying mutant PfCRT. Finally, we tested other drug combinations, including lidocaine + chloroquine (CQ), lidocaine + ALLN (a cysteine protease inhibitor), artemether (ATM) and two calcium channel blockers (nicardipine and cinacalcet), and ATM and lumefantrine (AL; a clinically used ACT) to investigate the roles of PfCRT and PfMDR1 in response to these combinations. Although our results were based on IC_50_ measurements that were different from those of DPC or the Ring-stage Survival Assays (RSA)^32^, our study provides important information on how parasite genetic background affects drug transport and metabolism, and therefore may affect DPC/RSA among parasites with the same or similar K13 mutations. A specific combination of changes in genes in drug transport, metabolism, degradation, and target binding is likely required for the development of a clinical DPC phenotype as observed in Southeast Asia.

## Results

### Compounds with correlated responses to DHA are linked to *pfmdr1 and/or pfcrt*

Previously, we screened a library consisting of 2,816 compounds approved for human or animal use and investigational drugs against 61 *P. falciparum* isolates and linked a large number of the compounds to *pfmdr1* and *pfcrt*^28^,^33^. Further analysis of the molecules we screened previously identified a group of 53 compounds with significantly positive correlation (correlation coefficient R > 0.7 and *P*-value < 10^−8^) to those of DHA in parasite responses (Table 1)^28^. Among the 53 compounds were ART derivatives such as artenimol (dihydroartemisinin) and artemetero (artemether) as well as those antimalarial drugs that were known to have similar response profiles to that of ART such as mefloquine, halofantrine and lumefantrine. Scatter plots of IC_50_ values of 61 field isolates for selected compounds showed good linear correlation between DHA and the compounds (Supplementary Fig. 1). The observations suggest that DHA and these compounds are likely metabolized through similar pathways or are transported by the same molecules such as PfCRT and PfMDR1. Indeed, 13 (24.5%) of the compounds could be significantly linked to PfMDR1 in the progeny of two genetic crosses (Dd2 × HB3 and 7G8 × GB4), which were further confirmed using PfMDR1 allelic exchanged parasites (Table 1). Additionally, 6 of the 53 (11.3%) compounds were associated with PfMDR1 in the field isolates. Similarly, 37 of the 53 compounds (69.8%) were significantly (*P* < 6.0 × 10^−5^) associated with PfCRT having false discovery rates (FDR) smaller than 5.0 × 10^−3^, including DHA and artemether (Table 1). These observations suggest that PfMDR1 and/or PfCRT play a role in the metabolism or transport of these compounds either individually or together. The results are also consistent with various studies showing association of specific PfMDR1 allele (7G8 allele) and copy number with response to ART/DHA^13^,^16^,^17^,^19^, although the role of PfCRT in DHA response has not been as clearly established as that of PfMDR1 previously. Furthermore, a recent GWAS study to identify makers associated with ART DPC phenotype found a significant association between increased *in vivo* ART DPC and mutant *pfcrt*^34^, suggesting that *pfcrt* may play a role in the decreased ART susceptibility phenotype.

### Correlated responses between Ca^2+^/Na^+^ channel blockers and DHA

**Figure 1:**
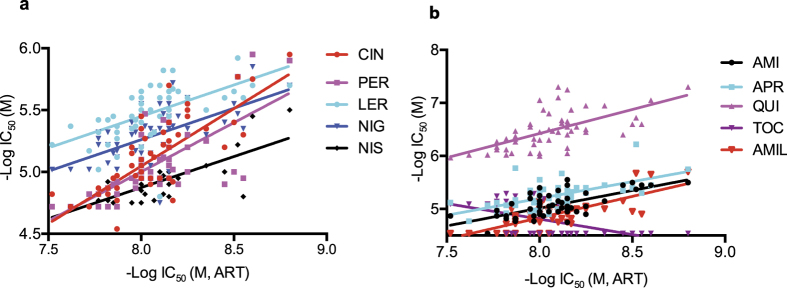
Scatter plots of parasite responses to calcium channel (**a**) and sodium (**b**) blockers against dihydroartemisinin (DHA). Half maximal inhibitory concentrations (IC_50_) from each of 61 field *Plasmodium falciparum* isolates were plotted. Each color represents a compound as indicated: CIN, cinacalcet (correlation coefficient R = 0.765); PER, perhexiline (R = 0.618); LER, lercanidipine (R = 0.598); NIG, niguldipine (R = 0.568); NIS, nisoldipine (R = 0.757); AMI, amiodarone (R = 0.674); APR, aprindine hydrochloride (R = 0.595); QUI, quinidine (R = 0.602); TOC, tocainide (R = −0.595); and AMIL, amiloride (R = 0.738). These analyses were based on raw data in our previously study^28^.

Using K-means and Dunn’s Index to generate an activity clustering without any prior assumptions about the types of expected activities to be observed, we clustered ART with other 16 compounds from the full set of 2816 compounds we screened previously, again including mefloquine, halofantrine and lumefantrine^28^. Interestingly, three of the compounds (lercanidipine hydrochloride, lasalocid sodium and niguldipine) were Ca^2+^ channel blockers or associated with Ca^2+^ fluxes, representing significant (*P* = 0.02, Fisher’s exact test) enrichment of Ca^2+^ channel blockers (Supplementary Table 1). The observation of clustering multiple channel blockers prompted us to search for additional Ca^2+^ and Na^+^ channel blockers within the library we screened previously and investigated whether the parasite responses to the channel blockers were correlated with response to ART. We identified 50 Ca^2+^ and 20 Na^+^ channel blockers (Supplementary Tables 2, 3 and 4) and found that many of the channel blockers had response patterns highly correlated to that of ART among the field isolates (Fig. 1). Among the Ca^2+^ channel blockers, 39 (80%, excluding ART) had correlation coefficients ranging from 0.324 to 0.803 with significant *P*-values (*P* < 0.02) (Supplementary Table 3). Similarly, 11 of the 20 Na^+^ channel blockers (55%) had R-values = 0.275–0.738 at *P* < 0.05 (Supplementary Table 4). The interactions of these ion channel blockers with ART and its derivatives were further corroborated in our recent large-scale combination screening study^35^. These observations suggest that various Ca^2+^ and Na^+^ channel blockers may also interfere with ART transport and/or metabolism.

### Confirmation of interactions of DHA and Ca^2+^/Na^+^ channel blockers

**Figure 2:**
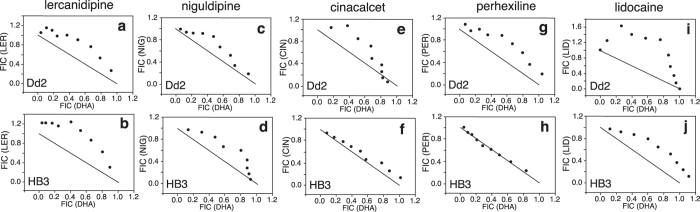
Isobolograms showing interactions of dihydroartemisinin (DHA) and selected calcium and sodium channel blockers for *Plasmodium falciparum* Dd2 and HB3 strains. (**a**,**b**) lercanidipine; (**c**,**d**) niguldipine; (**e**,**f**) cinacalcet; (**g**,**h**) perhexiline; (**i**,**j**) lidocaine. FIC, fractional inhibitory concentration. Ratios of 1.0:0 (DHA:blocker), 0.9:0.1, 0.8:0.2, 0.7:0.3, 0.6:0.4, 0.5:0.5, 0.4:0.6, 0.3:0.7, 0.2:0.8, 0.1:0.9, and 0:1.0 were used, where 1.0 equals 4–16 fold of the half maximal inhibitory concentration (IC_50_) of each compound. ΣFIC values are listed in Supplementary Table 8.

To determine if Ca^2+^/Na^+^ channel blockers interact with ART, we performed isobologram analysis on several channel blockers against dihydroartemisinin (DHA), including four Ca^2+^ blockers (perhexiline, cinacalcet, lercanidipine and niguldipine) and the Na^+^ blocker lidocaine that were readily available from commercial suppliers. Results from the isobologram analysis showed that these compounds were mostly antagonistic with DHA in Dd2 and HB3 parasites, except for cinacalcet and perhexiline that were additive in HB3 (Fig. 2). These results suggest that some Ca^2+^/Na^+^ channel blockers may interact differently with ART in distinct parasite lines, possibly influenced through the transport activities of PfCRT and/or PfMDR1 because 12 of the Ca^2+^ and Na^+^ channel blockers and several ART derivatives were also significantly associated with or linked to PfCRT and/or PfMDR1 (Supplementary Tables 1, 3 and 4).

### Parasite responses to Na^+^ channel blocker lidocaine mapped to *pfcrt*

**Figure 3:**
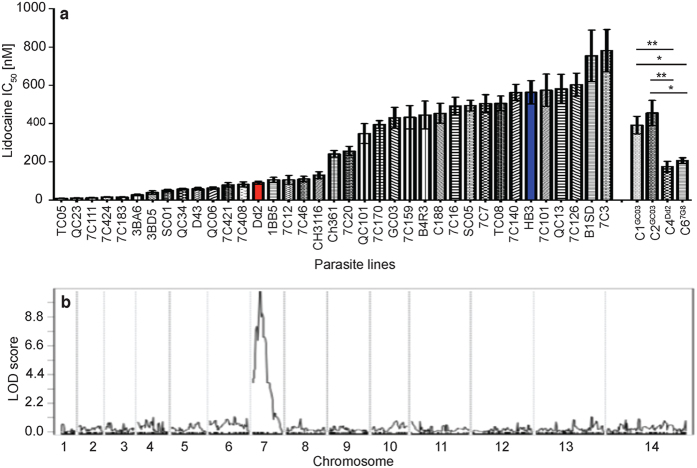
Plots of half maximal inhibitory concentration (IC_50_) of progeny and parents of the Dd2 × HB3 cross and scores of logarithm (base 10) of odds (LOD) in response to lidocaine. (**a**) IC_50_ values and standard deviations from three independent tests. Dd2 and HB3 are the parents, and the rest are progeny. C1^GC03^, C2^GC03^, C4^Dd2^, and C6^7G8^ are *pfcrt* allelic replaced parasites. C1^GC03^ and C2^GC03^ had wild type *pfcrt* (allelic exchange control parasites); C4^Dd2^, Dd2 *pfcrt* allele; C6^7G8^, 7G8 *pfcrt* allele. Shown for IC_50_ assays are the Mean ± SEM, conducted in at least three independent experiments, see Supplementary Table 5 for values. (**b**) Plot of LOD scores of parasite response to lidocaine analyzed using microsatellite markers described previously^38^. Qtl analysis was as described in the Methods section. t-test, *P < 0.05; **P < 0.01.

Comparison of the IC_50_ values of the Ca^2+^ and Na^+^ channel blockers between the five parents of the three *P. falciparum* genetic crosses showed that the 7G8 parasite was more sensitive to many of the blockers than 3D7 and GB4 (Supplementary Tables 3 and 4), which allowed genetic mapping of the determinants playing a role in responses to the compounds. Because the majority of the channel blockers associated with or linked to PfCRT were Ca^2+^ channel blockers, here we further investigated whether PfCRT or PfMDR1 could be linked to Na^+^ channel blocker lidocaine that could be obtained easily from commercial supplies. We first tested Dd2, HB3, 7G8 and GB4 responses to lidocaine using a 96-well SYBRGreen assay^36^,^37^. The results showed relatively large differences between Dd2 and HB3 in response to lidocaine (IC_50_ = 91.2 ± 8.2 μM for Dd2; IC_50_ = 563.4 ± 60.7 μM for HB3; Supplementary Table 5). The large difference in lidocaine IC_50_ value between Dd2 and HB3 and the antagonist relationship with DHA (Fig. 2i,j; Supplementary Table 8) prompted us to further investigate the genetic determinant(s) affecting parasite response to lidocaine. We measured the IC_50_ values for 23 progeny from the Dd2 × HB3 cross in response to lidocaine (Fig. 3a and Supplementary Table 5) and performed QTL analysis of the IC_50_ values against genome-wide microsatellite markers^38^. A peak with LOD score = 10.8 on chromosome 7 was identified, linking the response to lidocaine to markers on or near *pfcrt* (Fig. 3b). These results suggested that *pfcrt* plays a role in parasite response to lidocaine. *Pfcrt* was also previously linked to another channel blocker—verapamil^39^.

### PfCRT mediated altered drug-drug interaction

To confirm that *pfcrt* was indeed responsible for the differential activity of lidocaine, we tested the responses of parasites with genetically modified PfCRT alleles (Supplementary Table 6), which have been previously reported^40^. We tested two parasites derived from GCO3 that had their wild type PfCRT allele replaced with the CQR alleles of the Dd2 or 7G8 parasite, respectively. The IC_50_ values of GCO3 parasites engineered to carry the mutant PfCRT alleles of Dd2 or 7G8 (C4^Dd2^ or C6^7G8^) were approximately two-fold lower than IC_50_ values of control CQS GCO3 transformed parasites (C1^GCO3^ or C2^GCO3^, both allelic control parasites that retain the GCO3 PfCRT allele) (Fig. 3a and Supplementary Table 5). These results confirm that PfCRT plays a role in lidocaine response, possibly acting to transport the compound.

**Figure 4:**
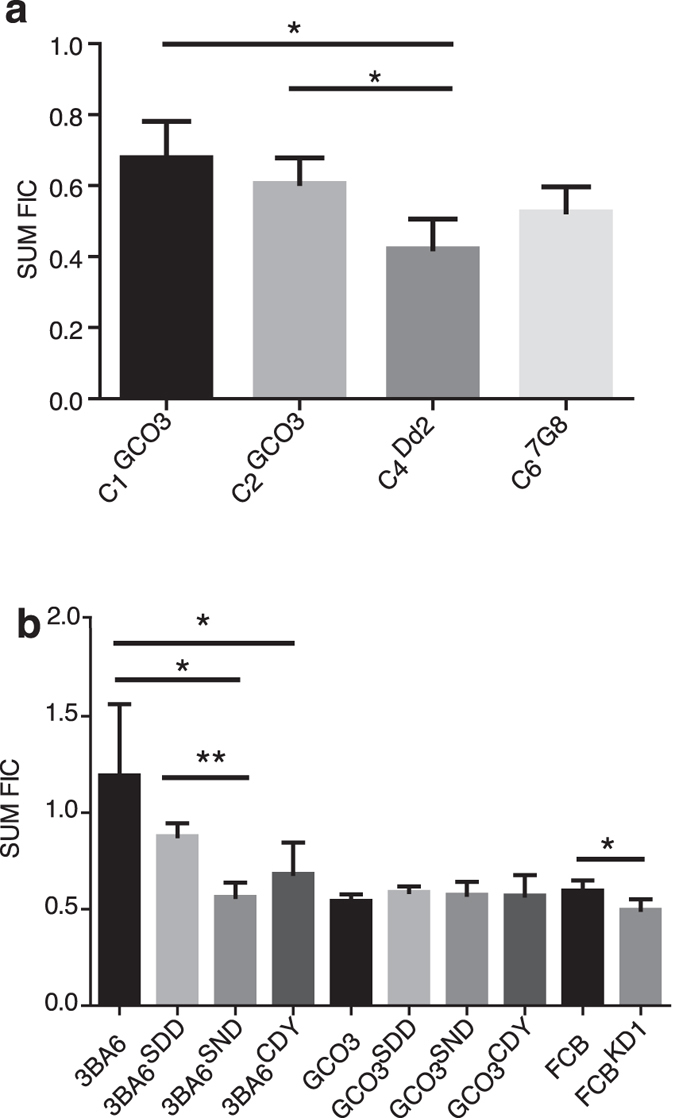
Responses of *pfcrt* and *pfmdr1* allelic exchange parasites to dihydroartemisinin + lidocaine (DHA + LID) combination. (**a**) Sum of fractional inhibitory concentrations (ΣFIC) of *pfcrt* allelic exchanged parasites in response to DHA + LID combination analyzed using isobolograms. The GCO3 parasite has a wild type *pfcrt*. C1^GCO3^ and C2^GCO3^ are both allelic exchange control parasite lines retaining the wild-type *pfcrt* locus, and C4^Dd2^ and C6^7G8^ are allelic exchange parasites with *pfcrt* replaced with the Dd2 or 7G8 allele, respectively. (**b**) Sum of fractional inhibitory concentrations (ΣFIC) of *pfmdr1* allelic exchanged parasites in response to DHA + LID combination analyzed using isobolograms. Indicated are the amino acids encoded at position 1034, 1042 and 1246. GCO3 and 3BA6 have the mutant SDD PfMDR1 allele; FCB has the wild type SND PfMDR1 allele. Shown for isobologram assays are the Mean ΣFIC ± SD, with at least three independent experiments. Note 3BA6^CDY^ was only significantly different from 3BA6, not the 3BA6^SDD^ allelic control parasite. Because both 3BA6 and 3BA6^SDD^ had the same SDD PfMDR1 allele, the lack of significance for both control parasites and the 3BA6^CDY^ modified line indicated that transgenic modification of the line was confounding the phenotype, precluding a clear conclusion about these comparisons; t-test, *P < 0.05; **P < 0.01.

As current antimalarial therapies are almost exclusively ACTs, we also determined if the drug-drug interaction between lidocaine and DHA was also modulated by variant PfCRT alleles. We assessed parasite response to various concentrations of lidocaine and DHA and calculated the summary of fractional inhibitory concentration (ΣFIC values or SumFIC). FIC is the minimum inhibitory concentration (MIC) of drug in combination divided by the MIC of drug acting alone, and the ΣFIC = FIC_A_ + FIC_B_ for drug A and B expresses the extent of the interaction^41^. We found that GCO3 parasites harboring the Dd2 PfCRT allele (C4^Dd2^) had a significant decrease in ΣFIC value, with a ΣFIC value 0.42 ± 0.08 compared to the allelic exchange control parasite C1^GCO3^ with a ΣFIC value of 0.68 ± 0.1 (*P* = 0.0285) (Fig. 4a and Supplementary Table 7). The results suggest that mutations in the PfCRT can influence the pharmacodynamic interaction of DHA and lidocaine.

### Effects of PfMDR1 alleles and copy number on DHA and lidocaine interaction

Another known drug transporter, PfMDR1, has also been shown to play a role in parasite responses to various drugs^16^,^18^. We therefore investigated the effects of specific amino acid substitutions and copy number variation in the *pfmdr1* gene mediating altered drug interaction. Again we used previously described allelic exchanged isogenic parasites either in the GCO3 or 3BA6 genetic backgrounds, including: the S1034C/N1042D/D1246Y (CDY) triple mutant PfMDR1 that is prevalent in South America (represented by 7G8 parasite), the 1034S/1042N/1246D (SND) as present in the wild type CQS 3D7 line, and the allelic exchange control parasites, GCO3^SDD^ or 3BA6^SDD^ (1034S/N1042D/1246D)^16^. The 3BA6 parasite also possesses the CQR K76T PfCRT mutation. In addition, we tested isogenic parasites with two functional copies of *pfmdr1* (FCB^(SND)^) and a genetically modified variant with a single functional copy (FCB^KD1^)^18^. The PfCRT and PfMDR1 genotypes of these parasites are listed in Supplementary Table 6. Our results showed significant differences in lidocaine-DHA interaction in the 3BA6^SND^ and FCB (both have the same mutant PfCRT) transgenic parasites harboring variant PfMDR1 alleles or copy number. Specifically, the significant differences between the pairs of 3BA6 vs 3BA6^SND^, 3BA6^SDD^ vs 3BA6^SND^, 3BA6 vs 3BA6^CDY^ showed that substitutions of amino acids in PfMDR1 could influence lidocaine-DHA interaction; whereas the significant difference between FCB vs FCB^KD1^ demonstrated that copy number variation in *Pfmdr1* could also affect the response to the drug combination (Fig. 4b and Supplementary Table 7). Both 3BA6 and 3BA6^SDD^ had the SDD alleles, but only the 3BA6 line (not the 3BA6^SDD^ control line) was significantly different from 3BA6^CDY^, suggesting that the introduction of the plasmid to replace its own allele might also influence the parasite response slightly. However, all the PfMDR1 variants in the GCO3 background (with wild type PfCRT) had similar drug-drug interactions with a ΣFIC ≤ 0.7. The results suggest that amino acid substitutions or copy number variation in PfMDR1 can also affect DHA and lidocaine interaction, but only in the modified lines with mutant PfCRT genetic background (3BA6 or FCB).

### Interactions of lidocaine and compounds known to interact with *pfcrt*

We also evaluated drug-drug interactions between lidocaine and compounds that putatively possess mechanisms of action involving the digestive vacuole using Dd2 and HB3 parasites. Significant differences in ΣFIC were found for both lidocaine + chloroquine (CQ) and lidocaine + ALLN (a cysteine protease inhibitor) between the parasites, with ΣFIC of 0.664 ± 0.13 for Dd2 and 1.272 ± 0.14 for HB3 (*P* = 0.035) in lidocaine + chloroquine combination, and ΣFIC 0.713 ± 0.05 for Dd2 and 1.209 ± 0.13 for HB3 (*P* = 0.021) in lidocaine + ALLN combination, respectively, suggesting that *pfcrt* and/or *pfmdr1* may play a role in these interactions (Supplementary Table 8). No significant difference was found for lidocaine + DHA nor lidocaine + E64 (a cysteine peptidases inhibitor), with a predicted weak antagonistic interaction found for each line.

### Drug-drug interaction between artemether (ATM) and channel blockers

**Figure 5:**
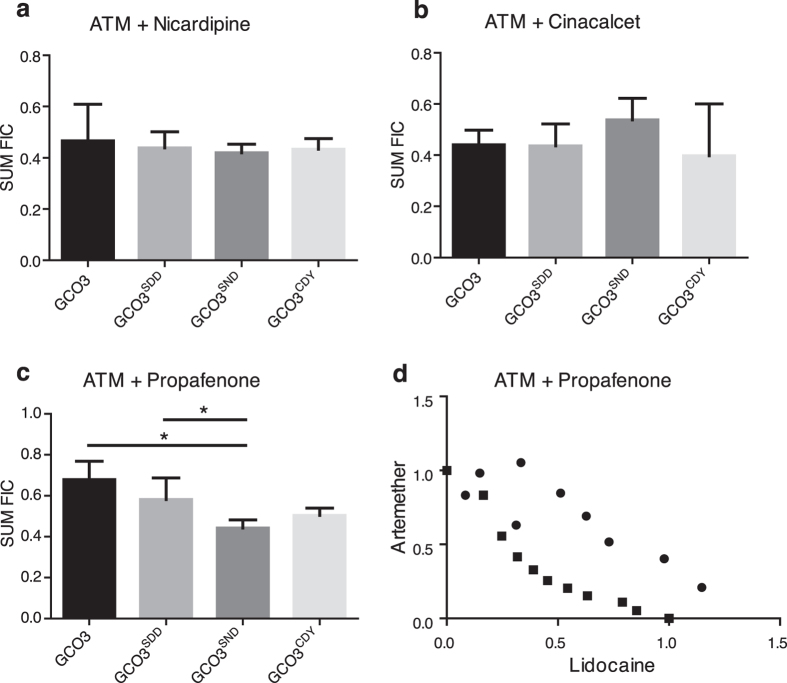
Responses of *pfmdr1* allelic exchanged parasites to various artemether (ATM) and channel blocker combinations (Mean ΣFIC ± SD). (**a**) ΣFIC of *pfmdr1* allelic exchanged GCO3 parasites in response to ATM + nicardipine. (**b**) ΣFIC of *pfmdr1* allelic exchanged GCO3 parasites in response to ATM + cinacalcet. (**c**) ΣFIC of *pfmdr1* allelic exchanged GCO3 parasites in response to ATM + propafenone. (**d**) Representative isobologram results for ATM + propafenone; circles, GCO3 (SDD PfMDR1); boxes, GCO3^SND^ (wild type PfMDR1 allele); *P < 0.05 (t-test, with at least three independent experiments).

As both the efficacy and drug-drug interaction of lidocaine and DHA are individually modulated by variant *pfcrt* and/or *pfmdr1* alleles and copy number, we hypothesized that the interactions of other compounds found to have correlated responses with ART against 61 *P. falciparum* isolates might also be modified by substitutions in PfMDR1. To test this hypothesis, we assessed interactions of ATM with two calcium channel blockers (nicardipine and cinacalcet) and a sodium channel blocker (propafenone). We found no significant modulation of the interaction between ATM and nicardipine or cinacalcet under GCO3 genetic background (Fig. 5a,b; Supplementary Table 7), again suggesting requirement of mutations in both *pfcrt* and *pfmdr1*. However, we detected a significant (*P* = 0.0319) change in interaction between ATM and propafanone in parasite GCO3^SND^ (Fig. 5c,d), which is consistent with previous mapping results linking propafanone DCP to *pfmdr1*^28^.

**Figure 6:**
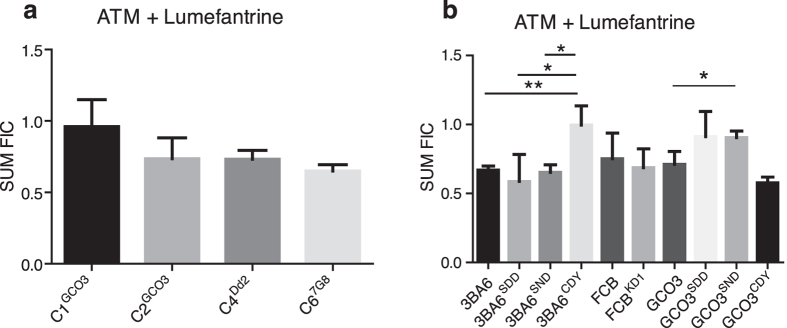
Responses of *pfcrt and pfmdr1* allelic exchanged parasites to Artemether (ATM) and lumefantrine (LUM) combination (Mean ΣFIC ± SD). (**a**) ΣFIC of *pfcrt* allelic exchanged GCO3 parasites in response to ATM + LUM combinations. (**b**) ΣFIC of *pfmdr1* allelic exchanged GCO3 parasites in response to ATM + LUM combinations. Shown are the ΣFIC values ± SD of at least three independent experiments, see Supplementary Table 7 for values. Note GCO3^SND^ was only significantly different from GCO3, but not the GCO3^SDD^ allelic control parasite having the same SDD PfMDR1 allele due to large variations in repeats, indicating that transgenic modification of the lines was confounding the phenotype, precluding a clear conclusion about these comparisons. t-test, *P < 0.05; **P < 0.01.

In addition to ion channel blockers, we also evaluated the interaction between ATM and lumefantrine (AL), a clinically utilized ACT, that has been shown to have correlated responses to ART derivatives^28^. There were no significant modulations of interaction between AL in the PfCRT-modified GCO3 lines (Fig. 6a; Supplementary Table 7). However, we found strain-specific modulations of interactions of AL in the PfMDR1-modified lines in the 3BA6 genetic background (Fig. 6b; Supplementary Table 7). The results again suggest the importance of mutations in *pfcrt* and *pfmdr1* on the observed pharmacodynamic interaction phenotype or the importance of mutant PfCRT in affecting PfMDR1 function in response to the compounds.

## Discussion

The emerging threat of ART resistance in Southeast Asia, and possibly other regions^2^,^42^,^43^,^44^,^45^,^46^, underscores the need to further understand ACT efficacy and the effects of drug-resistance determinants on the drug-drug interaction of combination therapies. Although the specific mechanism(s) by which ARTs exert their antimalarial activity remain contentious^47^, most studies support a model that the activity of ARTs results from the formation of potentially toxic heme-adducts or generation of free radicals that alkylate and oxidize proteins and lipids in parasitized erythrocytes^48^,^49^,^50^. Recent studies with a fluorescent redox indicator demonstrate a dose-dependent increase in oxidative stress with ART treatment, supporting that ARTs act, at least in part, by perturbation of the parasite redox state^3^. Furthermore, these studies demonstrate that inhibition of hemoglobin uptake, or hemoglobin digestion within the parasite food vacuole decreased the activity of endoperoxides^3^. In addition, previous studies demonstrate decreased ART susceptibility in recombinant parasites that have an altered *pfmdr1* allele or copy number variation^16^,^17^,^18^,^19^,^51^,^52^. Together, these observations support the digestive vacuole as an important site of activation of ARTs and possibly also as a location of cytocidal activity.

This study demonstrated that the DV-membrane resident transporters, PfCRT and PfMDR1, could mediate altered susceptibility to diverse compounds. QTL mapping of differential lidocaine response between Dd2 and HB3, supported by data from PfCRT recombinant isogenic parasites, showed that PfCRT could modulate parasite susceptibility to this compound. Further experimentation demonstrated that PfCRT and PfMDR1 could also modulate the pharmacodynamic interaction of DHA and lidocaine in certain genetic backgrounds. Although we found no significant differences in modulation of the DHA-lidocaine pharmacodynamic interaction between Dd2 and HB3 parasite lines (Supplementary Table 8), there were significant differences between Dd2 and HB3 in response to lidocaine + CQ and lidocaine + ALLN, suggesting a mechanism of action involving the DV (Supplementary Table 8). Taken together, these data support that the pharmacodynamic interaction of compounds whose mode of action involve the DV can be perturbed by altered PfCRT and/or PfMDR1 variants.

QTL mapping identified a locus on chromosome 7 that was significantly linked to altered lidocaine susceptibility in the Dd2 × HB3 progeny. Involvement of PfCRT in modulating this phenotype was confirmed using PfCRT allelic exchanged parasites with either Dd2 (C4) or 7G8 (C6) genetic background. However, the combination of DHA + lidocaine was only significantly altered in allelic exchange parasites with the Dd2 (C4) allele of PfCRT, suggesting that altered drug interaction may be a result of interaction between specific PfCRT (mutant) and PfMDR1 alleles. Recent metabolomic analysis comparing C2^GCO3^, C4^Dd2^, and C6^7G8^ parasites demonstrated that there was altered hemoglobin catabolism as measured by altered levels of hemoglobin peptide abundance between these isogenic lines^53^ providing support for altered DV biology between the C4^Dd2^ and C6^7G8^ parasites. Although PfCRT can modulate lidocaine drug susceptibility, possibly by altering lidocaine flux directly, altered pharmacodynamic drug-drug interaction may be influenced by PfCRT through secondary effects such as metabolite transport, which is also impacted by PfMDR1.

Although previous studies have demonstrated that PfMDR1 is able to modulate the susceptibility to ART and numerous ion channel blockers^16^,^28^, this study is the first to show that PfMDR1 can modulate the pharmacodynamic interactions of ATM with various ion channel blockers. The ATM + propafenone interaction was significantly altered only in the PfMDR1 GCO3^SND^ parasite (1034S, 1042N, 1246D; wild-type allele) compared to GCO3 with the SDD allele (1034S, N1042D, 1246D) and allelic exchanged GCO3^SDD^ control parasites. This would again suggest a complex pharmacodynamic interplay between each drug combination and the parasite genetic/biochemical background, with the resident DV transporters PfCRT and PfMDR1 playing a prominent role.

The complex interactions were also highlighted by our assays with AL, a clinically relevant drug combination. Based on a large scale matrix screen of 13,910 combinations against multiple *P. falciparum* lines, we found that the AL gave distinct interaction scores for Dd2 and HB3, with DBSumNeg values against Dd2 < −3 and those against HB3 > −3, suggestive of a synergistic interaction in Dd2, but not in HB3^35^. Interestingly, the PfMDR1 allelic exchanged parasites were divergent in how the DV transporter modulated the drug combination: In the 3BA6 background, the CDY allele produced an additive effect compared to a synergistic interaction in the control SDD lines and SND allelic exchanged parasite; in the GCO3 background, no significant difference was found for the modified parasites. The results again suggest that the pharmacodynamic phenotype produced by the drug combination is strongly influenced by the genetic background of the parasites, possibly a result of the different PfCRT alleles.

Although our results demonstrate that altered PfCRT and PfMDR1 mutations can modulate drug-drug pharmacodynamics, the clinical implications of 1246Y mutation in modulating AL efficacy are unclear. From multiple clinical and *in vitro* laboratory studies there is evidence that AL selects for the 1246D allele of PfMDR1, which mediates greater resistance to both individual components of the ACT^13^,^16^,^54^,^55^,^56^. However, our results did not show any significant difference in allelic exchanged parasites with this allele for AL. Importantly, the previous methods used to generate *pfmdr1* parasites had hampered the generation of isogenic parasites with the 86N and 184F mutations that strongly associate with AL recrudescence^57^; newly developed genetic editing methods should enable the generation of these parasite lines for future testing.

Our data also showed shared mechanisms of parasite responses to ART/DHA and many Ca^2+^ and Na^+^ channel blockers. We provided evidence that both PfCRT and PfMDR1 played an important role in parasite responses to ART and its derivatives and to various channel blockers. Additionally, PfCRT and PfMDR1 can also modulate drug-drug interactions, affecting antimalarial combination efficacy against *P. falciparum*. For many combinations, the effects of PfMDR1 allelic variations on drug-drug interactions were significant only in parasites with mutant PfCRT alleles, suggesting functional linkage between these two DV transporters. Interestingly, in a recent study, 124 ART covalent binding targets were identified, including a calcium-bing protein, a calcium-dependent protein kinase, and two calcium-transporting ATPases (ATP4 and ATP6) as well as PfMDR1 and PfCRT^58^. The identification of PfMDR1, PfCRT, and calcium-transporting ATPases as ART-binding targets is consistent with our observations and supports our conclusion that PfMDR1, PfCRT and possibly some calcium-transporting ATPases play a role in ART transport and/or metabolism. Multiple processes can influence drug action and drug resistance, including pro-drug activation, drug transport, inhibition of drug targets, and drug degradation/clearance. Because anti-plasmodial activity of ART requires heme-derived iron produced in the DV, and ART-induced oxidative proteome damages mainly occur in the cytoplasm^3^, PfCRT and PfMDR1 may therefore play a role in transporting ART through the DV membrane. The antagonist relationships of DHA and many of the channel blockers we observed (Fig. 2) also suggest that ART derivatives and the channel blockers are likely competing for these transporters, which can be explored for formulating effective drug combinations. Although it will require additional investigations to incorporate these observations with the recent report of the gene with K13-propeller domain being a key determinant in delayed clearance phenotype^10^, this study provides important information that advances our understanding of potential ART transport through the DV membrane by PfMDR1 and PfCRT. Testing compounds such as Ca^2+^ channel blockers that compete with ART for transporting molecules (PfMDR1 and PfCRT) may lead to better drug combinations for treating malaria.

## Methods

### Parasite culture

Asexual, blood-stage parasites were cultured *in vitro* using standard conditions^59^. Briefly, parasites were maintained in 2% human O^+^ erythrocytes (Interstate Blood Bank, Memphis, TN) in RPMI-1640 medium (Life Technologies, Grand Island, NY) supplemented with 0.5% Albumax (Life Technologies), 24 mmol/L sodium bicarbonate, and 10 μg/mL gentamycin. Tissue culture flasks were incubated at 37 °C under a gas mixture of 5% CO_2_, 5% O_2_, and 90% N_2_. *Plasmodium falciparum* lines, the progeny clones of the Dd2 × HB3 cross, and the transgenic *pfcrt* and *pfmdr1* parasites were used as previously reported^40^,^60^,^61^,^62^. Briefly, the *pfcrt* modified parasites used were: C1^GCO3^ that has a recombinant *pfcrt* with only a single intron (intron 1), C2^GCO3^ that has the second round plasmid integration but maintains the wild type *pfcrt*, and C4^Dd2^ and C6^7G8^ that carry either Dd2 or 7G8 *pfcrt* allele, respectively^40^. The *pfmdr1* modified lines are either in the GCO3 or 3BA6 genetic backgrounds, with GC03 having a wild type *pfcrt* and 3AB6 possessing the mutant Dd2 *pfcrt* allele (CQR). The amino acid substitutions (haplotypes SDD, SND, or CDY) occur at PfMDR1 position 1034, 1042, and 1246^16^. Lastly, the FCB parasite has two copies of *pfmdr1*, and FCB^KD1^ has only one copy^18^. The PfCRT and PfMDR1 genotypes of these parasites are summarized in Supplementary Table 6. Cultures were screened for mycoplasma using the Universal Mycoplasma Detection Kit (ATCC, Manassas, VA).

### Drug solution preparation

All compounds used were purchased from Sigma-Aldrich (St. Louis, MO), except for dihydroartemisinin (DHA), which was purchased from AvaChem Scientific (San Antonio, TX). All compounds were solubilized in DMSO, except for CQ that was solubilized in distilled water.

### Proliferation assays and IC_50_ analysis

*In vitro* drug responses were measured using 72-hr SYBR Green staining assays as described previously with minor modifications^63^,^64^. Parasites were diluted to 0.5% to 0.8% final parasitemia with 2% final hematocrit. The diluted parasite culture (100 μL) was added to duplicate test wells in a 96-well plate containing 100 μL of the drug tested. IC_50_ values were determined by nonlinear regression analysis using Prism 5.0 software (GraphPad Software, San Diego, CA). Drug assays were performed on three to ten independent occasions.

### Isobologram analysis

Drug combination assays were performed as previously described with minor modifications^65^. Drugs were mixed in volumetric ratios of 1:0, 9:1, 8:2, 7:3, 6:4, 5:5, 4:6, 3:7, 2:8, 1:9, 0:1 with 1 representing four- to sixteen-fold the IC_50_ of each drug. Drug combinations were serially diluted two-fold prior to the addition of parasites. Parasite inhibition growth was determined using 72-hr SYBR Green staining assays as described previously^37^,^64^. Sum of fractional inhibitory concentration (ΣFIC; where a value > 1.1 is defined as an antagonistic interaction, ≤0.7 as a synergistic interaction and values between 1.1 and 0.7 are additive) values were calculated on the basis of IC_50_ values obtained per assay for each drug (FIC_50_ = IC_50_ of drug A when used in combination with drug B/IC_50_ of drug A when used alone; ΣFIC = FIC_A_ + FIC_B_ = C_A_/MIC_A_ + C_B_/MIC_B_). The mean FIC_50_ values were calculated from at least three independent assays. ΣFICs were compared for each line using an unpaired t-test.

### Quantitative trait loci (QTL) analysis

QTL linkage analysis was performed using mean IC_50_ values (at least three repeats) from drugs tested and genome-wide microsatellite markers^38^ using the J/qtl analysis program^66^.

### Correlation and statistical analysis

Pearson correlation coefficients (R-values) were calculated using the log IC_50_ values. *P*-values were calculated using the exact bivariate normal distribution; similar *P*-values were obtained using either permutation-based method or based on normal distribution.

## Additional Information

**How to cite this article**: Eastman, R. T. *et al.* PfCRT and PfMDR1 modulate interactions of artemisinin derivatives and ion channel blockers. *Sci. Rep.* **6**, 25379; doi: 10.1038/srep25379 (2016).

## Supplementary Material

Supplementary Table S1

Supplementary Table S2

Supplementary Table S3

Supplementary Table S4

Supplementary Table S5

Supplementary Table S6

Supplementary Table S7

Supplementary Table S8

## Figures and Tables

**Table 1:** Compounds highly correlated with dihydroartemisinin (DHA) in responses among 61 field isolates.

Name	R-value	*P*-value	Name	R-value	*P*-value
artenimol (DHA)$	0.8393	3.53E-16	imazalil sulphate*,$	0.7546	7.86E-12
dipyridamole*,$	0.8241	1.08E-15	gluconic acid, barium salt	0.7535	2.08E-11
podofilox$	0.8135	4.89E-15	sulconazole nitrate	0.7521	6.58E-12
glutathione$	0.8093	1.50E-14	tolterodina$	0.7520	6.65E-12
ergotamine d-tartrate^#,$^	0.8091	8.97E-15	pronethalol$	0.7515	2.51E-11
tert-butylhydroquinone	0.8059	1.15E-13	distamycin a$	0.7498	8.27E-12
econazole nitrate*,$	0.8059	1.37E-14	propafenone$	0.7483	9.58E-12
(s)-timolol$	0.8057	1.40E-14	verteporfin#	0.7443	1.12E-10
triclosan*,$	0.8038	3.05E-14	benzyl alcohol	0.7422	3.91E-11
oxiconazole nitrate$	0.8033	1.94E-14	17-allylamino-geldanamycin$	0.7419	2.68E-11
chlorobutanol$	0.7982	3.72E-14	bifonazole*	0.7401	3.19E-11
benperidol^#,$^	0.7940	6.25E-14	amiloride$	0.7384	1.24E-10
toxaphene$	0.7916	8.42E-14	dihydroergocristine^#,$^	0.7311	4.88E-11
bromocriptine^#,$^	0.7916	8.44E-14	rifampin^#,$^	0.7225	1.04E-10
artemisininum (ART)	0.7880	1.30E-13	vitamin k2	0.7213	1.69E-10
tegaserod$	0.7832	3.70E-13	miconazole#	0.7208	1.21E-10
rifapentine^#,$^	0.7780	6.72E-13	lumefantrine^#,$^	0.7182	2.20E-10
levamisole hydrochloride$	0.7702	1.58E-12	clotrimazole^#,$^	0.7173	2.37E-10
mefloquine hydrochloride#	0.7700	2.54E-12	tolperisone hydrochloride	0.7165	3.37E-09
anthraquinone$	0.7682	1.89E-11	carminomycin	0.7142	2.13E-10
artemetero (artemether)$	0.7663	1.52E-12	fluticasone propionate$	0.7116	2.34E-09
cefsulodin sodium salt$	0.7660	1.57E-12	zeaxanthin#	0.7090	5.81E-09
tioconazole*^,$^	0.7659	1.59E-12	pirarubicin$	0.7089	3.33E-10
cinacalcet hydrochloride^#,$^	0.7654	2.62E-12	aa-861$	0.7041	4.92E-10
halofantrine hydrochloride#	0.7637	3.11E-12	meclozine dihydrochloride	0.7016	1.38E-08
clorophene$	0.7587	1.94E-11	sulisobenzone	0.7005	2.62E-09
dextroamphetamine saccharate^#,$^	0.7563	4.31E-12			

Name, compound names; R-value, values of Pearson correlation coefficient. The R values were calculated using the log_10_IC_50_ values. The *P*-values were calculated using the exact bivariate normal distribution.

*Associated with *pfmdr1* at P = <5.7E-5.

^#^Linked to *pfmd1* in crosses and confirmed by allelic exchanged parasites.

^$^Associated with *pfcrt* at p < 6.03E-05 and FRD < 5.0E-03.

These results were compiled and analyzed based on data in our previous screening^29^.
